# Study protocol: Epigenetic variations associated to the conversion of schizophrenia in ultra-high risk adolescents, a 4-year follow-up study

**DOI:** 10.1192/j.eurpsy.2023.1320

**Published:** 2023-07-19

**Authors:** P. Navalón, Y. Cañada, A. García-Blanco

**Affiliations:** Psychiatry Department, Hospital La Fe, Valencia, Spain

## Abstract

**Introduction:**

Schizophrenia is a severe mental disorder characterized by negative (e.g., social withdrawal, apathy, anhedonia) and positive (e.g., hallucinations, delusions, disorganized behaviour) symptoms. Schizophrenia prodromal symptoms typically emerge during the adolescence. These symptoms consist of attenuated and/or intermittent psychotic symptoms and define the ultra-high risk (UHR) states of schizophrenia. Approximately, 30% of UHR individuals develop schizophrenia. It is important to find biomarkers associated with the conversion of schizophrenia in UHR individuals in order to develop preventive and therapeutic strategies, as well as to gain knowledge in the ethiopathology of the disorder. In this regard, epigenetic variations have been identified as potential biomarkers associated with the conversion to schizophrenia, but the lack of research prevent to draw final conclusions.

**Objectives:**

The objective of this communication is to report the study protocol of the research project named “Epigenetic variations associated with the conversion of schizophrenia in ultra-high risk adolescents”. Its aim is to analyze the epigenetic marks that predict the development of schizophrenia in UHR youths.

**Methods:**

A 4-year observational follow-up study will be conducted, assessing three groups of adolescents who have sought clinical hep: 1) UHR group composed of individuals who have developed schizophrenia at the end of the follow-up; 2) UHR group composed of individuals who did not develop schizophrenia at the end of the follow-up; 3) a group composed of non-UHR individuals. Epigenetic marks will be analyzed in the peripheral blood of all the participants each 6 months. Clinical (i.e., positive and negative symptoms, depressive symptoms, anxiety symptoms, global functioning, traumatic events, alcohol and other toxics consumption) and sociodemographic data (i.e., age, sex, migration status, ethnicity, socio-cultural status, academic achievements) will be also assessed through standardized questionnaires.

**Results:**

We hypothesize that specific epigenetic marks will predict the conversion of schizophrenia in the UHR group. Moreover, interactions between epigenetic variations and sociodemographic data will differentiate the three groups after the follow-up.

**Conclusions:**

The findings of this project will help to develop preventive and therapeutic strategies, as well as to gain knowledge in the etiopathological pathways of schizophrenia.

**Disclosure of Interest:**

None Declared

